# Hemotropic pathogens in aborted fetuses of domestic ruminants: transplacental transmission and implications for reproductive loss

**DOI:** 10.3389/fmicb.2025.1632135

**Published:** 2025-07-09

**Authors:** Daria Jurković Žilić, Šimun Naletilić, Željko Mihaljević, Ema Gagović, Silvio Špičić, Irena Reil, Sanja Duvnjak, Maja Zdelar Tuk, Adnan Hodžić, Relja Beck

**Affiliations:** ^1^Laboratory for Parasitology, Department for Bacteriology and Parasitology, Croatian Veterinary Institute, Zagreb, Croatia; ^2^Laboratory for Pathology, Department of Pathology, Croatian Veterinary Institute, Zagreb, Croatia; ^3^Laboratory for Bacterial Zoonoses and Molecular Diagnostics of Bacterial Diseases, Department for Bacteriology and Parasitology, Croatian Veterinary Institute, Zagreb, Croatia; ^4^Centre for Microbiology and Environmental Systems Science (CMESS), Division of Microbial Ecology (DoME), Department of Microbiology and Ecosystem Science, University of Vienna, Vienna, Austria

**Keywords:** hemopathogens, transplacental transmission, abortion, ruminants, molecular diagnostics, Croatia

## Abstract

**Objective:**

Hemotropic pathogens of the genera *Anaplasma*, *Babesia*, *Theileria*, and hemotropic *Mycoplasma* are significant infectious agents in domestic ruminants, most commonly associated with vector-borne transmission. However, their potential for transplacental transmission and their contribution to reproductive disorders remains poorly understood. This study aimed to investigate the presence of hemopathogens in aborted fetuses of cattle, sheep, and goats in Croatia, and to evaluate their potential role in transplacental transmission.

**Methods:**

Molecular analyses were conducted on tissue samples from 651 aborted fetuses collected between 2016 and 2019 as part of national abortion surveillance programs. PCR screening followed by sequencing were used to detect Anaplasmataceae, *Babesia*, *Theileria*, and hemotropic *Mycoplasma*.

**Results:**

Thirteen hemopathogens were detected in 94 of 651 fetuses (14.44%), including *Anaplasma marginale, Anaplasma ovis, Anaplasma phagocytophilum, Theileria orientalis, Theileria ovis, Theileria* sp. OT3*, Babesia ovis, Babesia canis, Babesia vulpes, Mycoplasma wenyonii, Mycoplasma haemobos, Mycoplasma ovis,* and *Mycoplasma haemominutum*. The highest infection rates were observed in cattle (17.27%) and sheep (15.85%), while goats showed significantly lower prevalence (5.3%). *A. marginale* and *A. ovis* were the most frequently detected pathogens in bovine and ovine fetuses, respectively. Hemotropic mycoplasmas were reported for the first time in Croatia, with the first Western Balkan record of ‘Candidatus *M. haemobos*’. Our study represents the first molecular documentation of a wide array of hemopathogens in aborted ruminant fetuses in Croatia, strongly indicating the possibility of transplacental transmission. The detection of species-specific patterns and the unexpected identification of protozoan species typically associated with canines highlight complex epidemiological dynamics.

**Conclusion:**

Vertical transmission of the detected pathogens may play a role in abortion in endemic regions and should be integrated into differential diagnostic protocols for reproductive failure investigations.

## Introduction

A wide range of pathogens can cause diseases in domestic ruminants, leading to substantial economic losses. Among these, hemopathogens, including genera such as *Anaplasma, Ehrlichia, Babesia, Theileria*, and hemotropic *Mycoplasma* represent a particularly important group (Neimark et al., 2001; Bock et al., 2004; Messick, 2004; Kocan et al., 2010; Rar et al., 2021; World Organisation for Animal Health [WOAH], 2021). These infections may be subclinical or range in severity from mild to fatal, depending on the specific pathogen involved and the host’s immune status. Clinical signs often include hemolytic anemia, fever, weight loss, lethargy, and jaundice. Beyond direct health impacts, such diseases contribute to decreased productivity (milk and meat yield) and reproductive disorders, including abortion. Although traditionally associated with tropical and subtropical climates, tick-borne diseases are increasingly affecting livestock in temperate, industrialized regions due to the geographic expansion of tick vectors (Eskezia and Desta, 2016). While ticks remain the primary vectors for most hemopathogens, transmission of hemotropic *Mycoplasma* species more commonly occurs via iatrogenic or mechanical routes, involving contaminated instruments or biting insects (Arendt et al., 2024). Alternative transmission routes, including intrauterine or transplacental transmission, have increasingly been recognized in recent years (Hornok et al., 2011; Niethammer et al., 2018; Andrade et al., 2024). While there is growing scientific interest, the epidemiological significance of transplacental transmission for hemopathogens remains poorly understood. However, emerging evidence suggests that several pathogens within this group may be transmitted vertically and may contribute to reproductive complications, including abortion (Henker et al., 2020).

Transplacental transmission has been confirmed for various *Theileria* species such as *Theileria equi* in horses (Allsopp et al., 2007; Georges et al., 2011; Françoso et al., 2018), *Theileria lestoquardi* in small ruminants (Zakian et al., 2014; Esmaeilnejad et al., 2018), and *T. annulata* in cattle (Sudan et al., 2015; Selim et al., 2021). In cattle, transplacental transmission of *T. orientalis* is also well documented (Lawrence et al., 2016; Swilks et al., 2017; Mekata et al., 2018), and experimental studies have demonstrated a 100% abortion rate in pregnant cows infected via tick bites (Baek et al., 2003), highlighting its potential as a reproductive threat. Similarly, vertical transmission of *Babesia caballi* in horses (Bartolomé del Pino et al., 2023) and *Babesia bigemina* and *Babesia bovis* in cattle has been reported (Grau et al., 2013; Costa et al., 2016), with neonatal fatalities associated with *B. bovis* (Henker et al., 2020).

Among *Anaplasma* species, *Anaplasma marginale* is the most extensively studied regarding transplacental transmission, with evidence from both experimental and longitudinal field studies (Norton et al., 1983; Potgieter and Van Rensburg, 1987; Ribeiro et al., 1995; Silva and Fonseca, 2014.; Silva et al., 2015). *Anaplasma phagocytophilum* has also been shown to cross the placenta in both cattle (Pusterla et al., 1997; Henniger et al., 2013) and sheep (Reppert et al., 2013; Stuen et al., 2018) and has been detected in 2.4 and 1.9% ovine and caprine abortion cases (Gouvias et al., 2024).

Although vertical transmission of hemotropic *Mycoplasma* is considered rare, molecular evidence suggests that it can occur (Fujihara et al., 2011). Candidatus *M. haemobos* and *M. wenyonii*, have been detected in aborted fetuses and neonatal calves from infected cows (Hornok et al., 2011; Girotto-Soares et al., 2016), indicating that vertical transmission may serve as a route of hemoplasma infection (Sasaoka et al., 2015; Niethammer et al., 2018).

In many cases of abortion, the underlying cause is not identified, as abortions often arise from multiple factors. However, infectious agents – particularly those requiring advanced molecular diagnostic tools – are frequently implicated (Moeller, 2001; Hecker et al., 2023). Given the scarcity of research on hemopathogens in aborted ruminant fetuses, the present study aimed to investigate their potential intrauterine transmission using molecular diagnostics in fetuses of cattle, sheep, and goats submitted as part of abortion surveillance programs in Croatia.

## Materials and methods

### Tissue sampling from aborted fetuses

This study analyzed tissue samples from 651 aborted fetuses of domestic ruminants, including 336 cattle, 183 sheep, and 132 goats. The fetuses were submitted by veterinary practitioners to the Croatian Veterinary Institute as part of national abortion surveillance programs conducted between 2016 and 2019. The primary aim of these programs was to monitor pathogens such as *Brucella* spp. (bacteriological testing according to World Organization for Animal Health [WOAH], 2022, Terrestrial Manual, Chapter Brucellosis), *Coxiella burnetii* (Berri et al., 2000), and *Chlamydia* spp. (Berri et al., 2009). Additionally, the presence of *Toxoplasma gondii* was assessed in sheep and goat fetuses, and *Neospora caninum* in cattle. Detection was performed using PCR and sequencing, as described by Hughes et al. (2006) and Jones et al. (2000). Tissue samples collected for molecular analysis included the liver, lungs, placenta, and lochia from cattle; the liver, lungs, and placenta from sheep; and the liver and lungs from goats. All necropsies were performed under strict biosafety conditions to prevent cross-contamination. Separate sets of sterile instruments were used for each fetus, and work surfaces were disinfected between each procedure. The simultaneous reception of multiple fetuses was uncommon. An official submission form provided by the attending veterinarian, containing farm origin and relevant epidemiological data, accompanied each sample.

### Molecular analysis

DNA was extracted from 20 mg of tissue (liver, lungs, placenta or lochia) using either the QIAcube automated DNA isolation system with the QIAamp DNA Mini Kit (Qiagen, Hilden, Germany) or, in high-throughput settings, the KingFisher™ Flex automated instrument (Thermo Fisher Scientific, Waltham, MA, United States) with the MagMAX™ CORE Nucleic Acid Purification Kit (Applied Biosystems).

All samples were screened by conventional PCR for the presence of *Babesia/Theileria*, *Anaplasmataceae*, and hemotropic *Mycoplasma* spp. Detection of *Anaplasmataceae* was performed using primers ehr16SR/ehr16SD targeting a 345 bp fragment of the 16S rRNA gene (Parola et al., 2000). Positive samples corresponding to *A. marginale, A. ovis*, or *A. capra* were further amplified using primers targeting an 842 bp fragment of the msp4 gene (Hornok et al., 2007). Samples positive for *A. phagocytophilum* were additionally screened for a 1,256 bp portion of the groESL operon (Petrovec et al., 1999). *Babesia* and *Theileria* species were detected using primers targeting a ~ 560 bp region of the 18S rRNA gene (Beck et al., 2009). Hemotropic *Mycoplasma* spp. were amplified using primers targeting a ~600 bp fragment of the 16S rRNA gene (Varanat et al., 2011). All primers used are listed in Table 1.

**Table 1:** Primers used for the molecular detection of hemotropic pathogens in aborted ruminant fetuses.

Pathogen	Primer	Sequence (5′–3′)	Target gene	Size (bp)	Reference
*Babesia*/*Theileria*	BAB1	GTCTTGTAATTGGAATGATGG	18S rRNA	560 bp	Beck et al. (2009)
	BAB2	CCAAAGACTTTGATTTCTCTC			
*Anaplasma/Ehrlichia*	EHR16SD	GGTACCYACAGAAGAAGTCC	16S rRNA	345 bp	Parola et al. (2000)
	EHR16SR	TAGCACTCATCGTTTACAGC			
*Anaplasma phagocytophilum*	HSRV	TCAACAGCAGCTCTAGTWG	groESL	1,256 bp	Petrovec et al. (1999)
	HS43	ATAGTYATGAAGGAGAGTGAT			
*Anaplasma marginale/A. ovis*	MSP43	CCCCGGATCCTTAGCTGAACAGGAATCTTGC	msp4	842 bp	Hornok et al. (2007)
	MSP45	GGGAGCTCCTATGAATTACAGAGAATTGTTTAC			
*Mycoplasma*	Myco322s	GCCCATATTCCTACGGGAAGCAGCAGT	16S rRNA	600 bp	Varanat et al. (2011)
	Myco938as	CTCCACCACTTGTTCAGGTCCCCGTC			

PCR reactions (20 μL) included 10 μL of GoTaq^®^ G2 Master Mix (Promega, Madison, WI, United States), 7.2 μL of nuclease-free water, 0.4 μL of each primer (10 pmol/μL), and 2 μL of template DNA. Positive controls (*A. marginale, A. phagocytophilum, B. divergens, T. orientalis*, and *M. wenyonii*) and DNase/RNase-free water was included as a negative extraction control in each run.

Amplified products were visualized using QIAxcel capillary electrophoresis (Qiagen), employing the QIAxcel DNA Fast Analysis Kit and standard alignment/size markers. PCR products were purified using the ExoSAP-IT™ PCR Clean-up Reagent (Applied Biosystems) and sequenced by Macrogen Europe BV (Amsterdam, Netherlands) using the corresponding primer sets.

Sequence data were assembled and edited using SeqMan Pro 17 and SeqBuilder Pro 17 (DNASTAR, Madison, WI, USA), and identities confirmed by BLAST analysis^1^.

### Statistical analysis

Statistical analysis was conducted using one-way ANOVA to assess differences in prevalence across species and tissues. In case where the assumptions of normality (as determined by Shapiro–Wilk test) and homogeneity of variance (Levene’s test) were not met, non-parametric Kruskal–Wallis test was employed.

## Results

**Figure 1:**
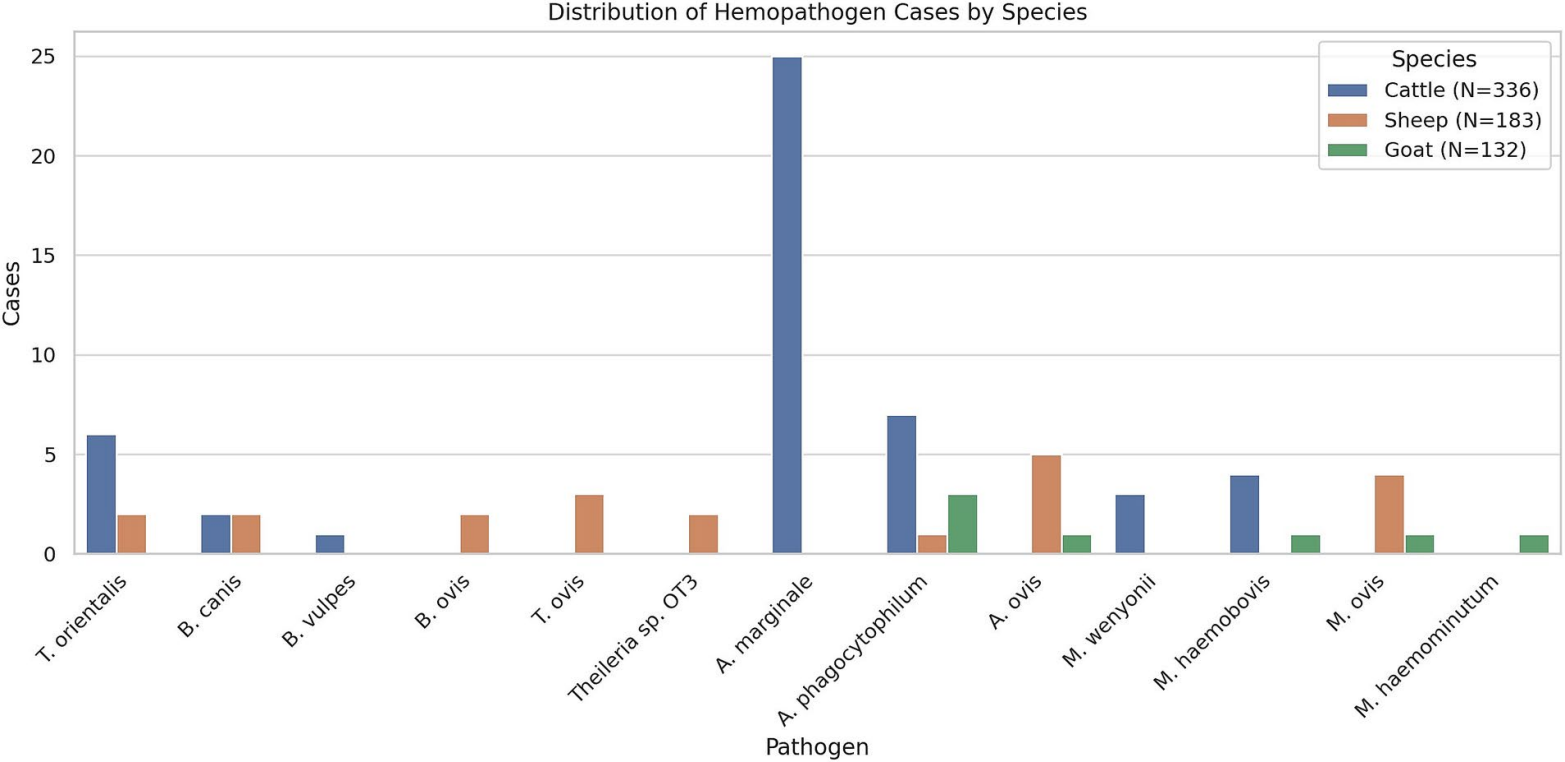
Distribution of identified hemopathogens in aborted fetuses of cattle, sheep, and goats. The bar chart shows the number of positive cases for each pathogen detected by molecular methods across the three ruminant species.

Out of 651 examined aborted fetuses, molecular evidence of infection with tick-borne or hemotropic pathogens was detected in 94 cases, corresponding to an overall prevalence of 14.44% (95% CI: 11.83–17.38). In this study, a total of 13 different pathogens were identified, including six piroplasm species, three *Anaplasma* species, and four hemotropic *Mycoplasma* species: *Babesia ovis, Theileria ovis, Theileria* sp. OT*3, Theileria orientalis, Babesia canis, Babesia vulpes, Anaplasma phagocytophilum, Anaplasma ovis, Anaplasma marginale, Mycoplasma ovis,* Candidatus *Mycoplasma haemominutum, Mycoplasma wenyonii*, and Candidatus *Mycoplasma haemobos* (Figure 1). Of these, 76 cases were single infections (11.67%, CI: 9.31–14.39), while co-infections were observed in 2.76% of cases (CI: 1.65–4.34). The highest prevalence was found in bovine fetuses, with 58 out of 336 infected (17.27%, CI: 13.38–21.74). Within this group, 48 out of 336 cases (14.29%, CI: 10.72–18.49) were found to be infected with a single pathogen, and 10 out of 336 cases (2.98%, CI: 1.44–5.41) were co-infected. In sheep fetuses, single infections were detected in 21 of 183 cases (11.48%, CI: 7.25–17.01), while co-infections occurred in eight of 183 cases (4.37%, CI: 1.91–8.43), resulting in an overall prevalence of 15.85% (CI: 10.88–21.96), with 29 of 183 cases (Table 2). When grouped by taxonomic affiliation, *Anaplasma* spp. were the most frequently detected pathogens identified in 59 out of 651 fetuses (9.06%; CI: 6.97–11.53), followed by piroplasms, which were detected in 32 fetuses (4.91%; CI: 3.39–6.87), and hemotropic *Mycoplasma* spp., which were confirmed in 22 cases (3.49%; CI: 2.20–5.23).

**Table 2:** Prevalence and coinfection rates of hemopathogens in aborted fetuses of cattle, sheep, and goats.

Pathogen	Cattle (N -336)	Sheep (N -183)	Goat (N -132)	Total (N -651)
*T. orientalis*	6 (1.79; 0.06–3.85)	2 (1.09%; 0.13–3.89)		8 (1.32%; 0.53–2.41)
*B. canis*	2 (0.6%; 0.07–2.13)	2 (1.09%; 0.13–3.89)		4 (0.61%; 0.17–1.57)
*B. vulpes*	1 (0.30%; 0.1–1.65)			1 (0.15%; 0–0.85)
*B. ovis*		2 (1.09%; 0.13–3.89)		2 (0.31%; 0.04–1.11)
*T. ovis*		3 (1.64%; 0.34–4.72)		3 (0.46%; 0.09–1.34)
*Theileria* sp. *OT3*		2 (1.09%; 0.13–3.89)		2 (0.31%; 0.04–1.11)
*A. marginale*	25 (7.44%; 4.87–10.79)			25 (3.84%; 2.50–5.62)
*A. phagocytophilum*	7 (2.08%; 0.8–4.24)	1 (0.55%; 0.01–3.01)	3 (2.27%; 0.47–6.50)	11 (1.69%; 0.85–3)
*A. ovis*		5 (2.73%; 0.89–6.26)	1 (0.76%; 0.02–4.15)	6 (0.92%; 0.34–2)
*M. wenyonii*	3 (0.89%; 0.18–2.59)			3 (0.46%; 0.09–1.34)
*M. haemobovis*	4 (1.19%; 0.33–3.02)		1 (0.76%; 0.02–4.15)	5 (0.77%; 0.25–1.78)
*M. ovis*		4 (2.19%; 0.60–5.50)	1 (0.76%; 0.02–4.15)	5 (0.77%; 0.25–1.78)
*M. haemominutum*			1 (0.76%; 0.02–4.15)	1 (0.15%; 0–0.85)
*T. ovis, A. ovis*		5 (2.73%; 0.89–6.26)		5 (0.77%; 0.25–1.78)
*A. ovis, M. ovis*		2 (1.09%; 0.13–3.89)		2 (0.31%; 0.04–1.11)
*A. phagocytophilum, M. ovis*		1 (0.55%; 0.01–3.01)		1 (0.15%; 0–0.85)
*T. orientalis, A marginale*	4 (1.19%; 0.33–3.02)			4 (0.61%; 0.17–1.57)
*A. marginale, M. wenyonii*	3 (0.89%; 0.18–2.59)			3 (0.46%; 0.09–1.34)
*T. orientalis, M. haemobovis*	1 (0.30%; 0.1–1.65)			1 (0.15%; 0–0.85)
*B. canis, A. marginale*	1 (0.30%; 0.1–1.65)			1 (0.15%; 0–0.85)
*M. wenyonii, A. marginale, T. orientalis*	1 (0.30%; 0.1–1.65)			1 (0.15%; 0–0.85)
Total	58 (17.27%; 13.38–21.74)	29 (15.85%; 10.88–21.96)	7 (5.30%; 2.16–10.62)	94 (14.44%; 11.83–17.38)

Seven pathogens were detected in bovine fetuses, including *T. orientalis, B. vulpes, B. canis, A. marginale, A. phagocytophilum, M. wenyonii,* and Candidatus *M. haemobos*. *Anaplasma* spp. had the highest prevalence, detected in 41 of 336 samples (12.2%, CI: 8.90–16.19), followed by piroplasms in 16 of 336 samples (4.76%, CI: 2.75–7.62) and hemotropic *Mycoplasma* in 12 of 336 samples (3.57%, CI: 1.86–6.16) (Table 2). The most frequently identified pathogen was *A. marginale*, which was detected in 34 of 336 samples (10.12%, CI: 7.11–13.87), followed by *T. orientalis*, which was found in 12 of 336 samples (3.58%, CI: 1.86–6.16). In addition, *A. phagocytophilum* and *M. wenyonii* were each identified in seven of 336 samples (2.08%, CI: 0.8–4.24), while Candidatus *M. haemobos* was detected in five of 336 samples (1.49%, CI: 0.49–3.44). Furthermore, *B. canis* was detected in three of 336 samples (0.89%, CI: 0.18–2.59), and *B. vulpes* in one of 336 samples (0.29%, CI: 0.01–1.65). Several co-infections were observed in bovine fetuses, including the combination of *T. orientalis* and *A. marginale* in four cases (1.19%), *A. marginale* and *M. wenyonii* in three cases (0.89%), and one case each involving *T. orientalis* and *M. haemobos, B. canis and A. marginale*, and a triple infection with *M. wenyonii, A. marginale*, and *T. orientalis*.

In sheep fetuses, the diversity of detected pathogens was the greatest among all studied animal species with the identification of eight different pathogenic agents, namely *B. ovis, T. ovis*, *Theileria* sp. OT3, *T. orientalis, B. canis*, *A. ovis, A. phagocytophilum,* and *M. ovis* (Table 2). Piroplasms were the most frequently detected pathogens, occurring in 16 of 183 samples (8.74%, CI: 5.08–13.81), followed by *Anaplasma* spp. in 14 of 183 samples (7.65%, CI: 4.25–12.50) and hemotropic *Mycoplasma* in seven of 138 samples (3.82%, CI: 1.55–7.72). *Anaplasma ovis* was identified as the most common pathogen, occurring in 12 of 183 samples (6.56%, CI: 3.43–11.17). This was followed by *T. ovis*, which was detected in eight of 183 samples (4.38%, CI: 1.91–8.43), and *M. ovis*, which was found in seven of 183 samples (3.82%, CI: 1.55–7.72). Other pathogens, including *T. orientalis, Theileria* sp. OT3, *B. canis, B. ovis*, and *A. phagocytophilum*, were each detected in two of 183 samples (1.09%, CI: 0.13–3.89). Several co-infections were observed in sheep, including *T. ovis* and *A. ovis* in five cases (2.73%), *A. ovis* and *M. ovis* in two cases (1.09%), and a single case with *A. phagocytophilum* and *M. ovis* (0.55%).

In goats, the overall prevalence of infection was lower than in cattle and sheep. Five pathogens were confirmed in goats: *A. ovis, A. phagocytophilum, M. ovis,* Candidatus *M. haemominutum,* and Candidatus *M. haemobos* (Table 2). Seven fetuses tested positive (5.30%; CI: 2.16–10.62), and only *Anaplasma* and hemotropic Mycoplasma species were detected. *Anaplasma phagocytophilum* was the most prevalent, confirmed in three fetuses (2.27%; CI: 0.47–6.50), while *A. ovis*, Candidatus *M. haemobos*, Candidatus *M. haemominutum*, and *M. ovis* were each identified in one fetus (0.76%; CI: 0.02–4.15). No piroplasms or co-infections were recorded in goats.

Mapping of positive cases revealed a widespread geographic distribution of hemopathogens across continental and coastal regions of Croatia in all three ruminant species. However, the density and diversity of pathogens varied by host species.

**Figure 2:**
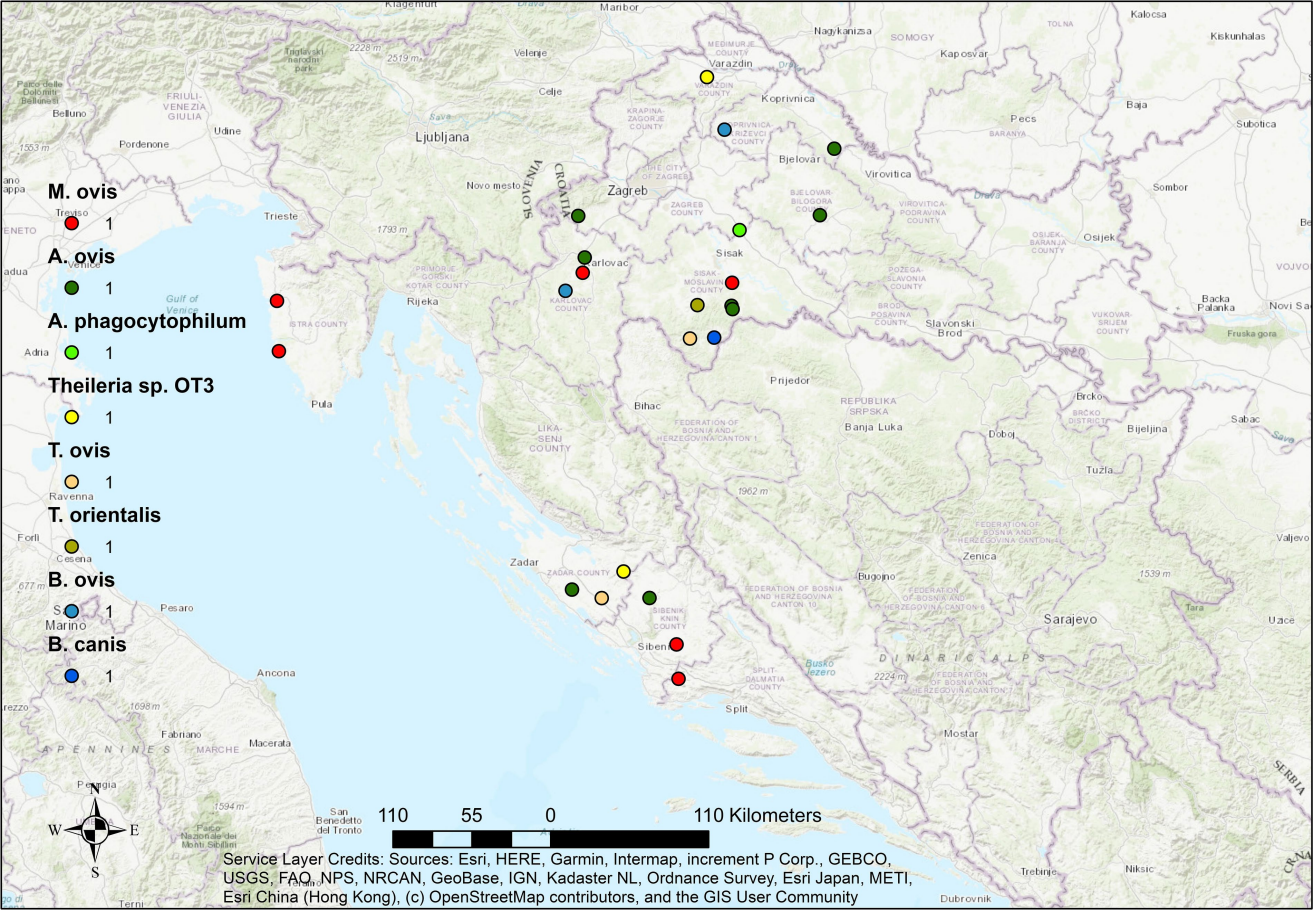
Geographical distribution of hemopathogens in aborted cattle fetuses in Croatia. Dots represent locations of individual cases. Color coding indicates haemopathogen, while size or density of symbols reflects number and diversity of pathogens detected at each site.

In cattle (Figure 2), most pathogen-positive cases clustered in central and eastern regions, with *A. marginale*, *T. orientalis*, and *M. wenyonii* detected most frequently. Several co-infections were also documented in this area, suggesting higher transmission pressure or improved detection due to population density and veterinary surveillance.

**Figure 3:**
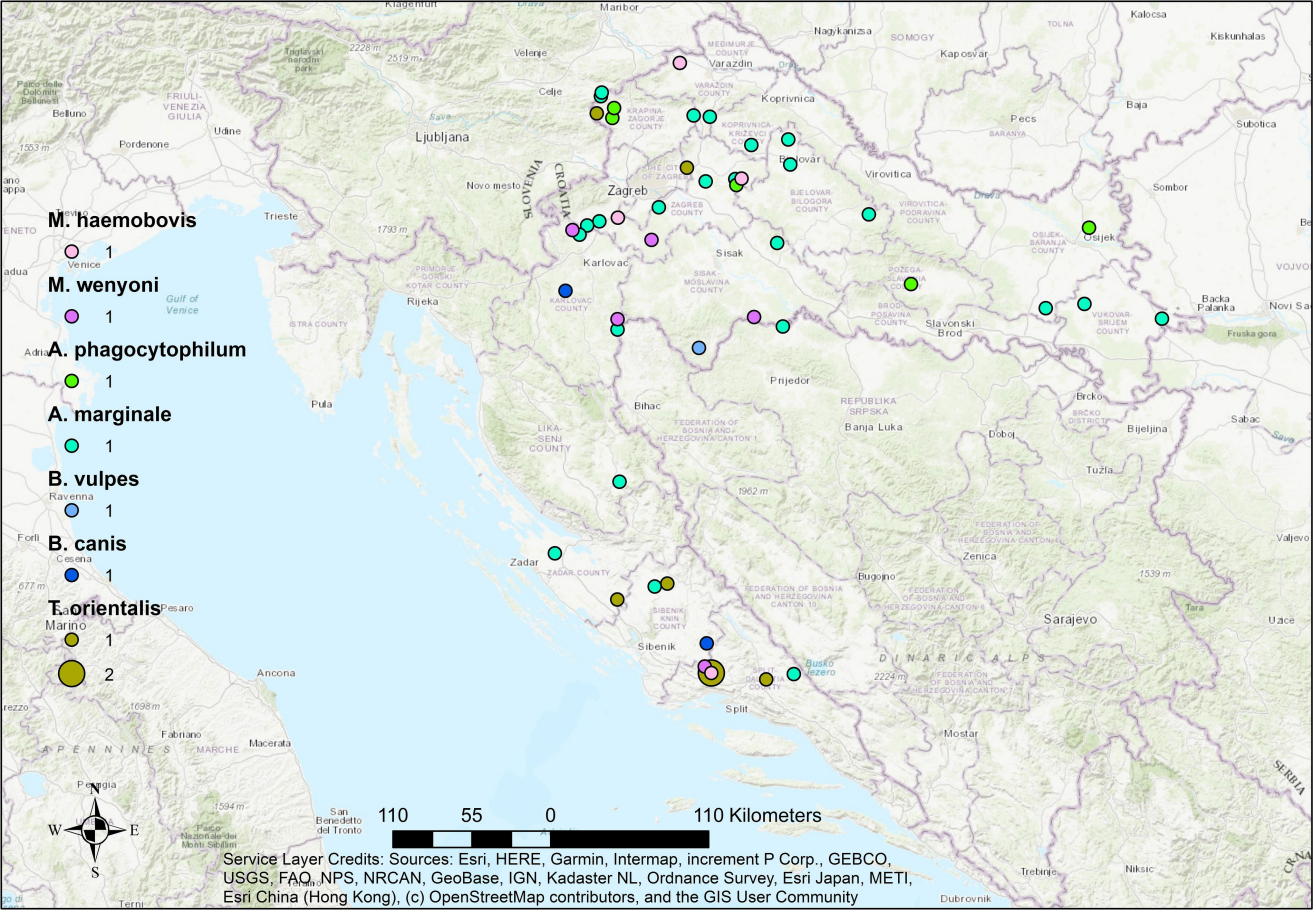
Geographical distribution of hemopathogens detected in aborted sheep fetuses in Croatia. Dots represent locations of individual cases.

In sheep (Figure 3), the spatial distribution of pathogens was more scattered but still showed notable clustering in northern and central regions. A high diversity of pathogens, including *T. ovis*, *A. ovis*, and *M. ovis*, was recorded, consistent with the observed high pathogen diversity in this species. Co-infections were observed in several localities, particularly where *T. ovis* and *A. ovis* overlapped, potentially indicating ecological compatibility or co-transmission by shared tick vectors.

**Figure 4:**
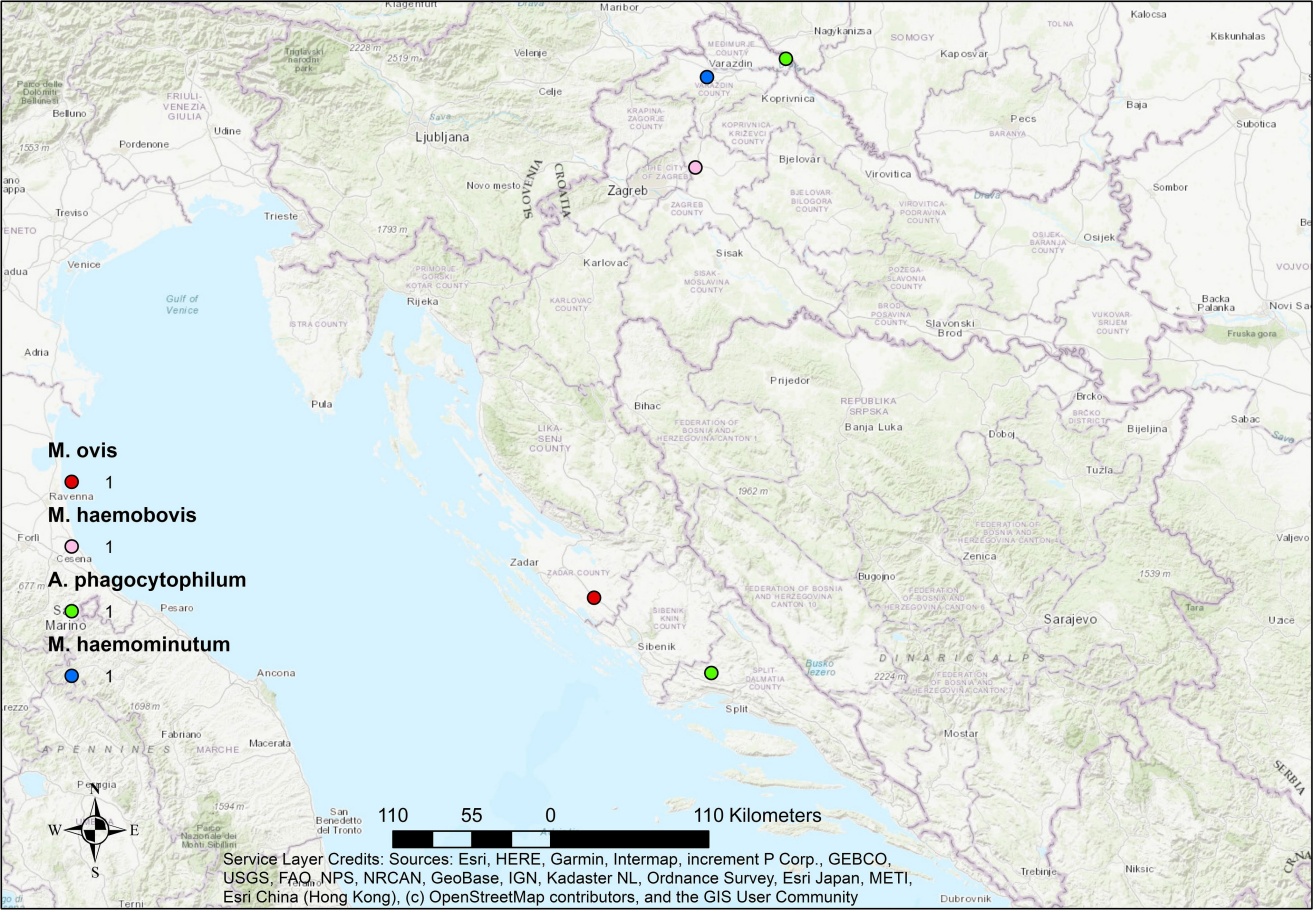
Geographical distribution of hemopathogens detected in aborted goat fetuses in Croatia. Dots represent locations of individual cases.

In goats (Figure 4), the geographic distribution of positive cases was the most limited, with fewer locations and lower pathogen diversity. Only *A. phagocytophilum*, *A. ovis*, and hemotropic *Mycoplasma* spp. were detected, predominantly in coastal and south-central regions. The limited spatial footprint may reflect both the lower number of submitted caprine fetuses and the species’ differing exposure risk or management practices. Overall, there were no strong differences in pathogen presence between coastal and continental regions, suggesting that vector presence and pathogen circulation are not strictly region-dependent.

In the present study, no co-infections with *C. burnetii, Brucella* spp., or *Chlamydia* spp. were detected in any of the examined animal species. In cattle, co-infection with at least one hemopathogen and *N. caninum* was confirmed in 14 out of 336 aborted fetuses, corresponding to a prevalence of 4.17% (95% CI: 2.30–6.89). In sheep, co-infection with *T. gondii* was confirmed in two out of 183 fetuses, corresponding to a prevalence of 1.09% (95% CI: 0.13–3.89) (Table 3).

**Table 3:** Coinfections of hemopathogens with *Neospora caninum* in cattle and *Toxoplasma gondii* in sheep fetuses.

Coinfections	Number of positive	Prevalence % (± 95% CI)
*N. caninum*, *A. marginale*	9/336	2.68% (1.23–5.02)
*N. caninum*, *M. wenyonii*	1/336	0.30% (0.1–1.65)
*N. caninum*, *M. haematobovis*	1/336	0.30% (0.1–1.65)
*N. caninum*, *A. phagocytophilum*	1/336	0.30% (0.1–1.65)
*N. caninum*, *A. marginale*, *M. wenyonii*	1/336	0.30% (0.1–1.65)
*N. caninum*, *M. wenyonii*, *A. marginale*, *T. orientalis*	1/336	0.30% (0.1–1.65)
*T. gondii, T. ovis, A. ovis*	1/183	0.55% (0.01–3.01)
*T. gondii, A. ovis*	1/183	0.55% (0.01–3.01)

In addition to prevalence estimates, statistical analysis was conducted to assess whether the likelihood of detecting hemopathogens in aborted fetuses differed significantly between host species. A chi-square test of independence showed a significant association between host species and infection status (*χ*^2^ = 11.38, *p* = 0.0034), suggesting non-uniform distribution of infection risk. Pairwise comparisons using Fisher’s exact test revealed that aborted cattle fetuses had significantly higher odds of testing positive for hemopathogens compared to goats (OR = 3.73, 95% CI: 1.65–8.39, *p* < 0.001). Similarly, sheep fetuses had higher odds of infection than goat fetuses (OR = 3.36, 95% CI: 1.43–7.93, *p* = 0.0038). However, no statistically significant difference was observed between cattle and sheep (OR = 1.11, 95% CI: 0.68–1.80, *p* = 0.71). These findings suggest that cattle and sheep may be at greater risk of hemopathogen-associated abortion compared to goats.

## Discussion

Hemopathogens represent an important group of infectious agents affecting domestic ruminants, particularly in tropical and subtropical regions. However, increasing evidence indicates that these pathogens may also be underrecognized in temperate areas, including Europe and Mediterranean Basin (Defaye et al., 2022). Despite historical reports of their presence in ruminants, recent molecular surveillance in this region has been limited (Kapo et al., 2025). Moreover, their potential role in reproductive disorders and fetal losses in ruminants has not been systematically investigated in Europe. This study provides novel insights into the transplacental occurrence of hemotropic pathogens in aborted ruminant fetuses in Croatia, using comprehensive molecular screening across three host species. Our findings reveal that infections with members of the genera *Anaplasma*, *Babesia*, *Theileria*, and hemotropic *Mycoplasma* are not only detectable in aborted fetal tissues but may also contribute to reproductive losses, including abortion.

The detection of *A. phagocytophilum, A. marginale*, and *A. ovis* was not unexpected, as these species have already been confirmed in Croatian ruminants (Jaarsma et al., 2019; Jurković et al., 2020; Šarić et al., 2022). Notably, *A. phagocytophilum* was the only hemopathogen detected in all three host species, albeit at low prevalence, consistent with findings from sheep flocks in Italy, Norway, Spain, the United Kingdom, and Greece (Jones and Davies, 1995; Garcia-Perez et al., 2003; Chianini et al., 2004; Lillini et al., 2006; Giudice et al., 2012; Gouvias et al., 2024), as well as goats in Greece (Chochlakis et al., 2020) and cattle (Van Loo et al., 2023). The low prevalence across species suggests a limited but potentially relevant role in reproductive disorders.

In contrast, *A. marginale* and *A. ovis* displayed a high degree of host specificity, being detected exclusively in cattle and small ruminants, respectively. These species were also the most commonly identified hemopathogens in their respective host species, both as single infections and in co-infections. High rates of transplacental transmission of *A. marginale* have been reported in Brazil and Venezuela, with prevalence in neonatal calves ranging from 10% to over 40% (Maldonado et al., 2012; Grau et al., 2013; Silva et al., 2014; Andrade et al., 2024). While vertical transmission is well documented, reports of abortion or fetal pathology directly linked to *A. marginale* remain scarce. Recent studies describing fetal lesions associated with *A. marginale* infection suggest that bovine anaplasmosis should be included in the differential diagnosis of fetal losses and neonatal mortality in endemic regions (Henker et al., 2020). Given that current study examined aborted fetuses, the observed association with *A. marginale* supports its potential role in reproductive failure, either as a primary cause or in conjunction with other pathogens.

In contrast, the association between *A. ovis* and fetal losses has not been established so far. In one experimental study, vertical transmission of *A. ovis* was not confirmed via direct parasitemia in neonatal lambs but was demonstrated by blood transfer to splenectomized recipients (Zaugg, 1987). Under natural conditions, *A. ovis* has been identified in neonatal elk calves (Hendrix et al., 2019), but not in domestic ruminants. Our detection of *A. ovis* in aborted sheep and a goat, both as a single agent and in co-infection with *T. ovis* and *M. ovis*, represents, to our knowledge, the first such finding suggesting possible intrauterine transmission and involvement in fetal pathology.

Interestingly, *A. bovis*, previously detected in deceased cattle in earlier study (Jurković et al., 2020), was not identified in any of the aborted bovine fetuses in this investigation. This observation may suggest that not all *Anaplasma* species possess the capacity for intrauterine transmission or contribute to fetal pathology.

Hemotropic *Mycoplasma* spp. were detected in domestic ruminants in Croatia for the first time in this study. Host-specific species were identified in their expected hosts, with *M. wenyonii* and Candidatus *M. haemobos* detected in cattle, and *M. ovis* in sheep and goats. Although intrauterine transmission of *M. ovis* has not been previously documented, parallels with other species—such as *M. haemolamae* in camelids—support the plausibility of vertical transmission (Tornquist et al., 2011).

Vertical transmission of bovine hemoplasmas is generally considered rare, but accumulating evidence suggests it is possible. Fujihara et al. (2011) first raised this possibility, while Hornok et al. (2011) demonstrated that 10.5% of neonatal beef calves born to infected dams were hemoplasma-positive. Subsequent studies by Sasaoka et al. (2015) and Niethammer et al. (2018) also supported the feasibility of vertical transmission. In one study by Tagawa et al. (2013), 14.1% of 71 dairy calves tested positive for bovine hemoplasmas; however, because blood sampling occurred up to a week after birth and neonatal animals were not sampled at birth, the route of transmission could not be definitively determined. Interestingly, *M. wenyonii* has previously been confirmed in neighboring Bosnia and Herzegovina (Stevanović et al., 2020), whereas the detection of Candidatus *M. haemobos* in our study represents the first confirmed case in the Western Balkan.

An unexpected finding was the detection of Candidatus *M. haemominutum* in a goat. Although typically regarded as a feline hemoplasma, this organism has also been identified in dogs (Zhuang et al., 2009) and wild canids such as wolves (Millán et al., 2018), raising the possibility of a broader host range and potential cross-species transmission.

The association between hemotropic *Mycoplasma* spp. and reproductive disorders remains poorly understood. However, our findings suggest that these organisms may contribute to fetal losses, either as sole infectious agents or in combination with other pathogens through synergistic co-infections.

Transplacental transmission of *T. orientalis* is of particular interest given the susceptibility of pregnant animals to abort. Prior studies indicate that vertical transmission of other *Theileria* spp. can occur in their respective hosts, including *T. equi* in horses (Phipps and Otter, 2004; Georges et al., 2011), *T. lestoquardi* in sheep (Zakian et al., 2014), and more rarely, *T. annulata* in cattle (Sudan et al., 2015). Experimental infection studies have demonstrated that transplacental transmission of *T. orientalis* can result in a 100% abortion rate in pregnant cows infected via ticks (Baek et al., 2003), while microscopic examinations in field settings in Japan suggested transplacental transmission at lower frequencies (Onoe et al., 1994). Conversely, a more recent study in New Zealand dairy herds experiencing *T. orientalis* outbreaks failed to confirm vertical transmission using highly sensitive qPCR assays (Lawrence et al., 2016).

In our study, it was identified in fetuses of both cattle and sheep, further supporting its abortifacient potential under field conditions (Baek et al., 2003; Swilks et al., 2017; Chisu et al., 2021). Additionally, the detection of *T. ovis* and *Theileria* sp. OT3 in aborted sheep fetuses expands the recognized diversity of *Theileria* species with the potential for intrauterine transmission. Alongside *Babesia ovis*, these findings indicate that a broader range of protozoan parasites may be implicated in small ruminant abortions than previously appreciated.

The presence of canine-origin piroplasms *B. canis* and *B. vulpes* in cattle and sheep, although unexpected, may reflect incidental spillover from dogs or wild canids, as both species have been previously reported in these hosts (Beck et al., 2009; Dežđek et al., 2010; Beck et al., 2017). Similar findings have been reported in Sardinia, where *Ehrlichia canis*, a canine tick-borne pathogen, was detected in aborted fetuses of sheep and goats (Chisu et al., 2021). However, the frequency of detection was substantially higher for *E. canis* than for canine piroplasms in the present study. Thus, it is likely that *B. canis* and *B. vulpes* detected here do not play a significant role in ruminant fetal pathology and represent incidental or non-pathogenic infections.

Importantly, no co-infections with classical abortifacient zoonotic pathogens such as *Brucella* spp., *C. burnetii*, or *Chlamydia* spp. were detected, suggesting that the hemopathogens identified in this study may act independently in causing fetal damage. However, co-infections with *Neospora caninum* in cattle and *Toxoplasma gondii* in sheep were observed, indicating the possibility of multifactorial causes of abortion.

The observed differences in pathogen prevalence and diversity underscore the complexity of vertical transmission dynamics in domestic ruminants. Notably, the significantly lower pathogen burden in goats suggests species-specific susceptibility. Although we cannot directly compute the probability of abortion due to infection (since we lack a control group of non-aborted fetuses), the observed infection prevalence in aborted fetuses may serve as a proxy indicator for potential vertical transmission and its relevance to reproductive loss.

## Conclusion

To date, limited but growing evidence supports the possibility of transplacental transmission of hemoparasites in cattle. However, data on small ruminants remain sparse. This study represents the most extensive molecular investigation in Europe involving all three major ruminant species, and clearly demonstrates that transplacental transmission of hemopathogens is possible and likely underrecognized. Our results highlight the need to include such pathogens in the routine diagnostic workup of abortions, especially in endemic areas. Long-term, multidisciplinary studies that incorporate detailed histopathological, molecular, and clinical data are necessary to comprehensively assess the role of hemopathogens in reproductive losses of domestic ruminants.

## Data Availability

The datasets presented in this study can be found in online repositories. The names of the repository/repositories and accession number(s) can be found in the article/supplementary material.

## Footnotes

^1^ https://blast.ncbi.nlm.nih.gov
